# Characterization of biocompatible scaffolds manufactured by fused filament fabrication of poly(3-hydroxybutyrate-*co*-3-hydroxyhexanoate)

**DOI:** 10.1098/rsos.211485

**Published:** 2022-04-06

**Authors:** Valentina Volpini, Alberto Giubilini, Lorenzo Corsi, Andrea Nobili, Federica Bondioli

**Affiliations:** ^1^ Department of Science and Methods in Engineering, University of Modena and Reggio Emilia, via Amendola 2, 42122 Reggio Emilia, Italy; ^2^ Centre En&Tech, Tecnopolo, p.le Europa 1, 42124 Reggio Emilia, Italy; ^3^ National Consortium of Material Science and Technology (INSTM), Via G. Giusti 9, 50121 Firenze, Italy; ^4^ Life Science Department, University of Modena and Reggio Emilia, 41125 Modena, Italy; ^5^ National Institute for Biostructures and Biosystems (INBB), Viale Medaglie d’Oro 305, 00136 Roma, Italy; ^6^ Department of Engineering Enzo Ferrari, University of Modena and Reggio Emilia, via Vivarelli 10, 41125 Modena, Italy; ^7^ Department of Applied Science and Technology (DISAT), Politecnico di Torino, Corso Duca degli Abruzzi 24, 10129 Torino, Italy

**Keywords:** bio-based scaffolds, cytocompatibility, fused filament fabrication, effective mechanical properties

## Abstract

We characterize poly(3-hydroxybutyrate-*co*-3-hydroxyhexanoate) (PHBH) scaffolds for tissue repair and regeneration, manufactured by three-dimensional fused filament fabrication (FFF). PHBH belongs to the class of polyhydroxyalkanoates with interesting biodegradable and biocompatible capabilities, especially attractive for tissue engineering. Equally, FFF stands as a promising manufacturing technology for the production of custom-designed scaffolds. We address thermal, rheological and cytotoxicity properties of PHBH, placing special emphasis on the mechanical response of the printed material in a wide deformation range. Indeed, effective mechanical properties are assessed in both the linear and nonlinear regime. To warrant uniqueness of the material parameters, these are measured directly through digital image correlation, both in tension and compression, while experimental data fitting of finite-element analyses is only adopted for the determination of the second invariant coefficient in the nonlinear regime. Mechanical data are clearly porosity dependent, and they are given for both the cubic and the honeycomb infill pattern. Local strain spikes due to the presence of defects are observed and measured: those falling in the range 70–100% lead to macro-crack development and, ultimately, to failure. Results suggest the significant potential attached to FFF printing of PHBH for customizable medical devices which are biocompatible and mechanically resilient.

## 1. Introduction

Sustainability concerns and environmental awareness have turned the scientific and industrial community’s attention to the exploration of bio-based materials whose performance parallels that of traditional petroleum-based plastics [1]. Among the available biopolymers, polyhydroxyalkanoates (PHAs) belong to the class of aliphatic polyesters whose applications are constantly growing, owing to their bio-origin and biodegradability [2]. Indeed, PHAs are naturally synthesized by microorganisms, prevalently bacteria, under unbalanced conditions of nutrients and other growth factors [3]. They represent a really broad family of thermoplastic polyesters whose actual mechanical and thermal properties may significantly vary according to the chemical structure of the polymer. In fact, they can be designed and synthesized targeting the desired expected properties for the intended application.

Recent studies highlighted remarkable potential for biomedical applications of PHA for tissue regeneration [4,5], controlled drug release [6,7] and vessel stenting [8], on account of its excellent cytocompatibility with the immune system, that prevents undesired toxic or inflammatory reactions [9]. Furthermore, PHA reveals a significant advantage over traditional scaffold materials, such as ceramics or metals, in that it is reabsorbable, meaning that it disintegrates while natural tissue regenerates, eventually disappearing at the end of the healing process [10].

Early investigations of biomedical applications of PHA addressed simple systems obtained by traditional manufacturing methods, such as solvent casting [11], salt leaching [12], thermally induced phase separation (TIPS) [13], emulsification [14] and electrospinning [15]. All these approaches suffer from the limitation of exerting little control over the structure development and operate at scales above a few hundreds of microns, which is clearly insufficient for extensive use in the biomedical field [16]. Besides, they require large amounts of harmful solvents, e.g., chloroform or dichloromethane.

Additive manufacturing (AM) is revolutionizing the biomedical sector, mainly in light of the high level of customization that it enables while, at the same time, keeping good control over complex structures and shapes without requiring cost- and time-expensive dies [17]. Most importantly, it allows tailoring each implant to the specific features of the recipient, thus achieving optimal compatibility [18]. To date, different approaches exist with regard to AM and tissue regeneration. In [19], the use of hydrogel-based bioprinted scaffolds is considered as a promising solution to produce complex geometries that accelerate the regeneration process. A very up-to-date review on the use of hydrogels in tissue engineering is given in [20]. Yet, hydrogels generally suffer from poor printability and weak mechanical performance, which demands the adoption of composite hydrogels with various organic and nano-organic fillers.

Among the extrusion-based AM techniques, fused filament fabrication (FFF) is probably the most common. In it, a thermoplastic polymer filament is melted, extruded through a nozzle, and then deposited in layer-by-layer fashion over a building platform. FFF allows printing different thermoplastic polymers with microscale resolution. Indeed, printing of conventional polymers, such as poly(acrylonitrile-*co*-butadiene-*co*-styrene) (ABS) [21], poly(lactic acid) (PLA) [22], polycaprolactone, [23,24] or poly(methyl-methacrylate), [25], has been thoroughly investigated and, in fact, this technology is commercially available and commonly used. In their pioneering work [23], Hutmacher *et al*. suggest FFF as the preferable manufacturing technique for scaffold fabrication on account of the possibility to tailor macroscopical mechanical performance through varying the internal infill, in terms of geometry and density, pore size and distribution. The role of FFF printing parameters in affecting the scaffold porosity is discussed in [26] for ABS. Similarly, in [22] authors numerically investigate mechanical response of different shapes of PLA scaffolds for bone implants.

Nowadays, a large part of the research endeavour is directed at developing unconventional polymers with original properties for novel applications. For FFF, a considerable technological barrier is constituted by the filament melting process prior to extrusion, which affects the polymer molecular weight, and it may cause an unsustainable drop in terms of mechanical properties. Indeed, this is the case of poly(3-hydroxybutyrate) [27]. Consequently, an important issue to consider when evaluating applicability of a new class of materials for FFF printing is adequate rheological behaviour and thermal stability [28,29]. Similarly, mechanical characterization of the printed material is still in its infancy, for it requires dealing with a thermally degraded, highly anisotropic and possibly inhomogeneous material.

Precisely these issues are addressed in this paper for poly(3-hydroxybutyrate-*co*-3-hydroxyhexanoate) (PHBH). This is a PHA copolymer constituted by two distinct monomers, namely 3-hydroxybutyrate and 3-hydroxyhexanoate (3HH), whose molar ratio can be used as a tailoring parameter for the final property of the copolymer. For example, by increasing the molar content of 3HH, there is an increase in the degree of crystallinity and elongation at failure [30]. This thermoplastic polymer is suitable for FFF printing and its thermo-mechanical properties may be tailored using different reinforcing agents such as cellulose nano-crystals [31]. In [27], different PHA polymers are compared, concluding that PHBH performs comparably to PLA. In fact, ‘PHBH exhibits excellent mechanical properties, no cytotoxicity and large proliferation of mouse embryonic fibroblast cells'. Also, PHBH is listed among the best candidates for FFF printable materials with reduced environmental footprint [32]. To the best of the authors’ knowledge, only a handful of recent papers have investigated FFF printability of PHBH, particularly in the field of three-dimensional printed biomedical scaffolds for bone regeneration [27,33]. Also, rigorous determination of the scaffold effective mechanical properties has not been attempted yet.

In this work, spotlight is set on (i) evaluation of thermal stability and rheological properties of PHBH, in order to exclude possible degradation of the biopolymer during the printing process; (ii) assessment of PHBH biocompatibility through direct and extraction tests; (iii) determination of effective mechanical properties of printed PHBH, by digital image correlation (DIC) and conventional testing protocols, in the linear and nonlinear regime and considering two different infill patterns; finally (iv) estimation of how well these properties describe the effective global response of the scaffolds. Specifically, effective mechanical properties are obtained directly by DIC analysis, while back fitting the numerical models is used only for the second nonlinear invariant.

The novelty of this work lies in its comprehensive approach, which encompasses the structural, biological and mechanical take on the material and its biomedical applications.

## 2. Experimental data

### 2.1. Materials

PHBH containing 11 mol% of hydroxyhexanoate is supplied by MAIP Group (Settimo Torinese, TO, Italy) in pellet form. PHBH pellets are oven-dried at 85°C overnight, then fed to a piston extruder (Rosand RH7, Netzsch GmbH, Germany) equipped with a capillary die with an orifice diameter of 1.8 mm to obtain filaments. Extrusion occurs at constant temperature and speed, respectively, 145°C and 8.5 mm min^−1^. Filaments with a diameter of 1.75 ± 0.05 mm are finally obtained.

### 2.2. Specimen design and fused filament fabrication printing

Specimens are designed with a three-dimensional CAD software (Autodesk Tinkercad) whereby stl format files are obtained. These are further sliced and transformed in gcode format (by the software Slic3r), which is then fed to a TREA Stilla3D FFF printer (Stilla3D, Italy), together with the PHBH extruded filament.

FFF printing produces

**Figure 1.**
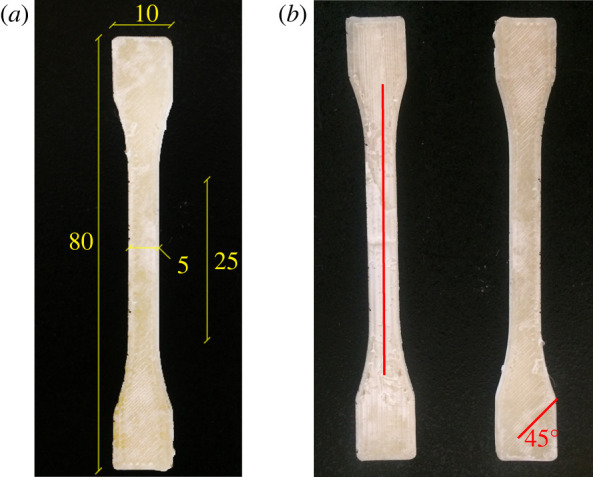
Dumb-bell specimen geometry (mm) (*a*) and infill structure (*b*).

— Solid (100% infill) cubic specimens for compression tests, with side *l* = 10 mm.
— 3 mm thick dumb-bell standard specimens for tensile testing as in [34], type 1 BA, illustrated in figure 1. Hundred per cent infill is realized at two distinct printing angles, namely 45° and 0° with respect to the specimen longitudinal axis, respectively, referred to as *diagonal* and *longitudinal infill*.
— Two families of cubic scaffolds with side *l* = 20 mm having different patterns for the internal structure: either rectilinear or honeycomb (cubic/honeycomb porosities, respectively). Infill parameters are selected so that the same quantity of polymer is fused and deposited for both families. Specifically, the slicing software calculates that a 1152 mm long filament is needed for each scaffold, regardless of the internal geometry. *Five specimens* are manufactured within each scaffold family (repetitions). The average scaffold mass is 3.17 ± 0.08 and 3.12 ± 0.13 g, respectively, for rectilinear and honeycomb porosity, which fact confirms that nearly the same amount of material is deposited.

For the sake of clarity, three-dimensional printing parameters are gathered in table 1.

**Table 1.** Three-dimensional printing parameters.

characteristic	unit	cubes	dumb-bell		scaffolds	
			long.	diagonal	cubic	honeycomb
nozzle temperature	°C	175	175	175	175	175
bed temperature	°C	60	60	60	60	60
nozzle diameter	mm	0.4	0.4	0.4	0.4	0.4
first layer height	mm	0.1	0.1	0.1	0.1	0.1
layer height	mm	0.33	0.33	0.33	0.25	0.25
perimeter	—	1	2	2	1	1
infill pattern	—	R	R	R	R	H
infill density	%	100	100	100	18	15
infill angle	°	45	90	45	45	45
infill speed	mm s^−1^	10	10	10	10	5
perimeter speed	mm s^−1^	5	5	5	5	5

### 2.3. Material characterization

#### 2.3.1. Thermal analyses

Thermal properties of PHBH have been evaluated by differential scanning calorimetry (DSC 2010, TA Instruments). Ten milligram samples are tested in nitrogen flow. In order to erase the previous thermal history, samples first undergo heating at 10°C min^−1^ from 25 to 200°C, followed by cooling at 20°C min^−1^ to −50°C. In the second thermal cycle, they are heated up again to 200°C at 10°C min^−1^. Cold crystallization temperature, *T*_cc_, melting temperature, *T*_m_ and glass transition temperature, *T*_g_ are determined from this second heating cycle.

Thermogravimetric analysis (TGA) is conducted on 6 mg PHBH samples in a STA 449 F3 Jupiter (Netzsch GmbH, Germany) device. Specimens are heated at the rate of 5°C min^−1^ from 50 to 600°C in air, with a air flow rate of 60 ml min^−1^.

Rheological behaviour of neat PHBH is determined through the rotational rheometer Anton Paar MCR 502, with a 25 mm parallel plate geometry and a gap of 0.5 mm at the constant temperature of 175°C. Flow curves under shear rates ranging from 10^−3^ to 10 s^−1^ are obtained through time-controlled measurements. Beyond the shear rate 10 s^−1^, samples leave the gap between the parallel plates.

#### 2.3.2. Cell culturing

BALB/3T3 clone A31-1-1 cells are provided by the Istituto Zooprofilattico Sperimentale (IZSBS, Brescia, Italy). Cell cultures are grown using Dulbecco’s Modified Eagle’s Medium (DMEM), supplemented with 10% FBS (Australian origin) and 0.5% (v/v) penicillin-streptomicin (Invitrogen, Italy) in standard conditions (95% humidity, 5% ${\mathrm{CO}}_{2}$, 37°C), and maintained in a subconfluent state (less than 80% confluence).

#### 2.3.3. Cytocompatibility

Prior to any cell culturing experiment, the material is sterilized with 99° ethanol and then UV-light for 30 min. Possible cytotoxicity of the biopolymer is assessed following ISO 10993-5 (direct contact) and ISO 10993-12 (extraction).

Direct contact tests are performed on a plate with 24 multiwells, each with surface area of 2 cm^2^ (Euroclone, Italy), by placing PHBH samples in the wells and directly above the cells for 24 h. The biopolymer mass (density *ρ* = 1.2 g cm^−3^) in each well is calculated according to the formula

2.1 w>ρAt,

where *w* indicates the sample weight and *ρ*, *A* and *t* are the density, the area required for the sample and the sample thickness, respectively. Parameters are set as to ensure that at least 10% of the well surface area is covered, i.e. a minimum surface of 20 mm^2^. After 24 h, cell viability is determined by adding 20 μl of 3-(4,5-dimethylthiazol-2-yl)-diphenyltetrazolium (MTT) (Sigma, Italy) solution (5 mg ml^−1^) and then incubating the cells at 37°C for 3.5 h. Next, the MTT solution is gently removed and 1 ml of acidic isopropanol (0.04 M HCl in absolute isopropanol) is added, until complete dissolution of the crystals. Cell viability is measured using a spectrophotometer (Multiscan FC Thermo Scientific) at 595 nm. Relative cell viability is normalized to the mean of the blank control medium. In addition, the morphology of 3T3 cells in contact with the polymer was fixed with PFA 4% at RT, washed and stained with 1 ml of GIEMSA (Sigma, Italy) 1 : 10 solution, and finally visualized in the optical microscope (Nikon Eclipse E100).

Extraction tests are performed to determine cytotoxicity of leachable materials from the PHBH specimens. Both biopolymer and latex, the latter being used as a positive cytotoxic control, were incubated in inert and sterile closed tubes at 37°C for 24 h in DMEM culture media at a concentration of 3 cm^2^ ml^−1^. After 24 h, 100 μl of pure extraction medium (EM, 100%) or after dilution with DMEM (final concentration of EM 50%, 25%, 10%), are added to pre-seeded 3T3 ($3\times 10^{5}\;\mathrm{cells}\;{\mathrm{cm}}^{-2}$) and incubated at 37°C and 5% ${\mathrm{CO}}_{2}$. Cell viability is assessed after 24, 48 and 72 h of continuous exposure with the aforementioned concentrations, by using the Counting Kit-8 (CCK-8) assay (Dojindo Laboratories, Kumamoto, Japan). In short, 10 μl of CCK-solution are added to each well and incubated for a period of 3 h at 37°C. Cell viability is measured at 450 nm using a multiplate reader Multiscan FC (Thermo Scientific, USA).

#### 2.3.4. Statistical analysis

All data are presented in terms of the mean ± 1 s.d. evaluated in at least two different experiments, each done in quadruplicates. One-way ANOVA analysis of variance with Dunnett’s post-test, or unpaired *t*-test, is performed to define statistical difference between the groups (Graph-Pad 6 Software Inc., San Diego, CA, USA). Values *p* < 0.05 are considered significant.

#### 2.3.5. Mechanical properties

The following tests are carried out to assess the mechanical properties of PHBH extruded filaments as well as of FFF scaffolds:

— Uniaxial compression tests on PHBH printed solid cubes. Following [23], specimens are tested under displacement control at 1 mm min^−1^. To account for the filament deposition direction, specimens are compressed either longitudinally (i.e. in the extrusion direction) or transversally. Six specimens are tested overall. The elastic modulus in compression, *E*_C_, is evaluated as the slope of the initial linear part of the stress–strain curve. Compressive strength at yield, *σ*_Y_, is defined as the stress occurring at the intersection point between the stress–strain curve and the line $\sigma =E_{C}(\varepsilon -1\text{\%})$.
— Tensile tests on PHBH printed dumb-bell specimens. Six dumb-bell specimens are printed as described in §2.2. The (mean) elastic modulus in tension, *E*_T_, ultimate tensile stress, *σ*_B_ and the tensile strain at failure, *ε*_B_, are determined from the stress–strain curves. Specifically, following [35], *E*_T_ is obtained as
  2.2 $$E_{T}=\frac{\sigma {|}_{\epsilon_{2}}-\sigma {|}_{\epsilon_{1}}}{\epsilon_{2}-\epsilon_{1}},$$
  where $\epsilon_{1}=0.5\text{‰}$ and $\epsilon_{2}=2.5\text{‰}$.
— Uniaxial compression tests on PHBH printed scaffolds with cubic and honeycomb porosity. Three specimens are tested within each porosity family at the displacement rate of 0.1 mm min^−1^.

Tests are performed under displacement control in a Instron 5567 electromechanical universal testing machine (UTM), equipped with a 30 kN load cell. Raw data are made available as electronic supplementary material.

**Figure 2.**
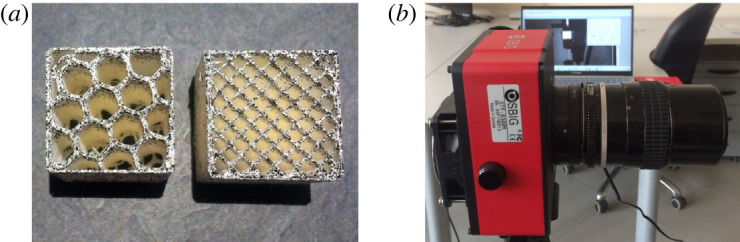
High-contrast pattern on FDM scaffolds with honeycomb/cubic porosities (*a*) and CCD camera (*b*).

*DIC analysis*. Along both tensile and compression tests, DIC is adopted to determine the displacement field in the specimen. For this, the specimen surface is painted with an ultra-fine high-contrast black/white speckle pattern, as shown in figure 2*a*. In order to mitigate glares and reflections, the specimen surface is illuminated by a diffuse light source (MI-LED Illuminator, Edmund Optics Ltd). Digital images have been acquired by a high-quality monochrome CCD camera (SBIG STF-8300m, see figure 2*b*) and finally post-processed by means of the commercial software GOM Correlate 2020. As we shall presently show, this analysis allows the experimental determination of Poisson’s ratio for the deposited material.

*Finite-element model*. Since the material behaviour of FFF printed scaffolds is rather complex and depends on many parameters (such as the printing process), *effective elastic properties* are determined instead. With a certain degree of approximation, these enable to design FFF printed scaffolds as if they were composed of homogeneous isotropic material. To this aim, numerical simulations are performed through a commercial FE software (COMSOL Multiphysics^®^) until best fit against experimental results is reached. Since best fitting is a strongly nonlinear procedure, multiple sets of best-fit parameters are common. To circumvent this difficulty, we rely on direct experimental determination for Poisson’s ratio and Young modulus in tension. Within the small deformation (linear) regime, we only apply best fitting to the determination of the effective Young modulus *E*_C_ in compression. In the linear regime, we adopt the classical isotropic Hooke Law (neo-Hookean compressive solids)

2.3 $$W=\frac{1}{2}G(I_{1}-3-2\ln J)+\frac{1}{2}k{\left(J-1\right)}^{2},$$

where *G* is the shear modulus, *k* the bulk modulus, *I*_1_ the first invariant of the Cauchy–Green deformation tensor and *J* indicates the determinant of the deformation tensor. In terms of the familiar Young’s modulus *E*_*C*_ and Poisson’s ratio *ν*, we have the well-known connections

$$G=\frac{E_{C}}{2(1+\nu )}\;\mathrm{and}\;k=\frac{E_{C}}{3(1-2\nu )}.$$

Since Poisson’s ratio reveals that the material is compressible, we extend our analysis to the finite deformation (nonlinear) regime by assuming a compressible Mooney–Rivilin form for the strain energy density

2.4 $$W=c_{1}(I_{1}-3)+c_{2}(I_{2}-3)+\frac{1}{2}k{\left(J-1\right)}^{2},$$

wherein *I*_2_ is the second invariant of the Cauchy–Green deformation tensor and *c*_1_ = *G*/2, *c*_2_ and *k* are constitutive material parameters. These have been determined from the linear regime, with the notable exception of *c*_2_, which is set by best fitting the stress–strain curve in scaffold compression tests, see §3.3.4.

## 3. Results and discussion

### 3.1. Thermal properties of poly(3-hydroxybutyrate-*co*-3-hydroxyhexanoate)

**Figure 3.**
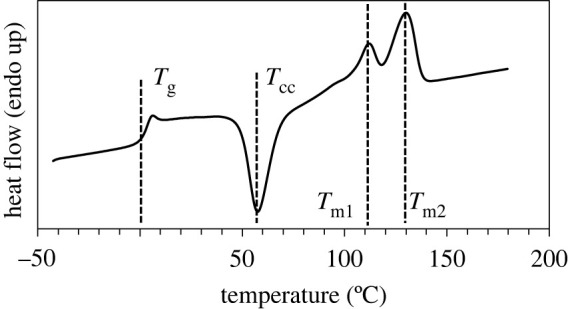
DSC curve of PHBH in the second heating scan: glass transition, *T*_g_, cold crystallization, *T*_cc_, and melting temperatures, *T*_m1_ and *T*_m2_, are clearly detected.

The thermal behaviour of PHBH, as determined by DSC analysis, is illustrated by the representative figure 3, obtained after having erased the previous thermal history of the material. As it clearly appears, PHBH displays glass transition, cold crystallization and multiple melting phenomena. Specifically, we observe glass transition at *T*_g_ = 2°C, cold exothermal crystallization at *T*_cc_ = 58°C, and two melting peaks: the first, *T*_m1_, at 112°C, and the second and final, *T*_m2_, at 130°C. This bimodal melting pattern, typical of polyesters, was already observed for this copolymer in [27,36]. This behaviour is due to the presence of crystals melting and reorganizing into new crystals with higher structural perfection, which are more stable and melt at higher temperature, namely *T*_m2_. As reported in [37], the first crystal form, melting at *T*_m1_, is characterized by the same structure as the second, more stable crystal form, melting at *T*_m2_, apart from showing smaller lamellar thickness.

**Figure 4.**
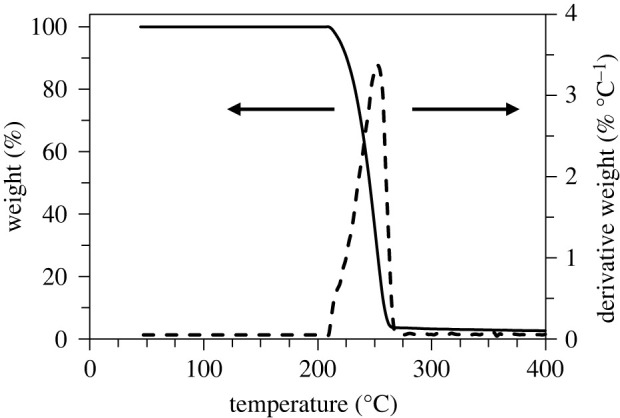
PHBH thermogravimetric analysis (solid) and its derivative (dashed curve): thermic degradation occurs beyond *T*_onset_ = 215°C.

In order to rule out any possible degradation of the biopolymer during the printing process, its thermal stability and rheological behaviour are hereinafter assessed. Figure 4 shows the results of a TGA (black solid line) and of its derivative (DTG, black dashed line). The TGA curve displays a distinct and rapid mass loss starting at the onset temperature for thermal degradation *T*_onset_ = 215°C, corresponding to an initial 2.5% weight loss. The maximum volatilization rate is attained at *T*_peak_ = 252°C, and it is referred to as the *peak decomposition temperature*. Such results are in agreement with, albeit slightly lower than, the corresponding temperatures shown in table 5 of [27]. Therefore, we can conclude that thermal degradation kicks in well beyond the assumed printing temperature of 175°C, so thus FFF printing does not significantly affect the material properties.

With the aim of evaluating the shear rate, $\dot{\gamma }$, experienced by the PHBH filament during the printing process, we follow [31] and relate the shear rate under scrutiny to that of a capillary system, according to the law

3.1 $$\dot{\gamma }=\frac{4v}{r},$$

in which *v* is the printing speed and *r* is the nozzle radius. By assuming 5 mm s^−1^ < *v* < 10 mm s^−1^ and *r* = 0.2 mm, we obtain the typical shear rate experienced by the melt polymer during extrusion through the nozzle, which occurs at $\dot{\gamma }$ from 100 to 200 s^−1^. Data were collected with a parallel plate rheometer with a shear rate up to 10 s^−1^. To extrapolate experimental viscosity values outside the measured range of shear rates, the empirical Bird–Carreau–Yasuda (BCY) model is used [38]

3.2 $$\eta (\dot{\gamma })=\eta_{\infty }+(\eta_{0}-\eta_{\infty }){\left[1+{\left(\lambda \dot{\gamma }\right)}^{a}\right]}^{(n-1)/a}.$$

**Figure 5.**
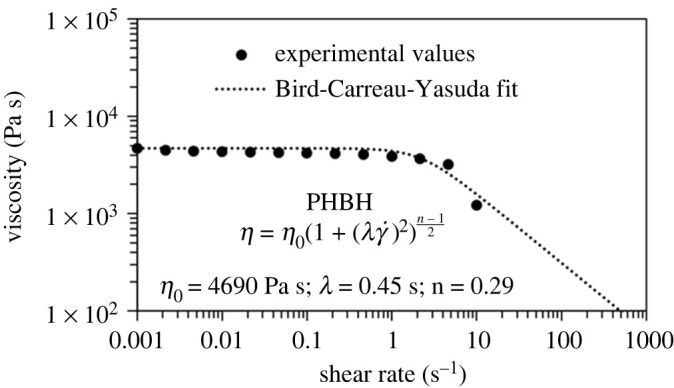
Experimental shear viscosity data (solid circles) of neat PHBH and theoretical fitting (dotted line) according to the BCY model (3.2). Experimental measurements were performed at the assumed printing temperature of 175°C.

In equation (3.2), *η*_0_ and *η*_∞_ indicate the zero and infinite shear rate viscosities, respectively, the latter being assumed 0, *a* is a dimensionless coefficient which is usually set to 2, *λ* is the relaxation time reflecting the onset shear rate of the shear-thinning behaviour, and *n* is the power-law index affecting the slope of the shear-thinning region (*n* − 1). According to the BCY model, the biopolymer behaves as a Newtonian fluid at low shear rates $\dot{\gamma }\ll \lambda^{-1}$, where viscosity plateaus at the constant value *η*_0_. By contrast, at large shear rates $\dot{\gamma }⪆\lambda^{-1}$, the material behaves as a power-law fluid and, consequently, it demonstrates shear thinning, i.e. it relaxes upon greater shear rates. Once parameters in (3.2) are determined from experimental data, figure 5 shows that actual experimental points demonstrate excellent agreement with the theoretical prediction. At the typical shear rate occurring in our FFF three-dimensional printing process (100−200 s^−1^), viscosity ranges from 310 to 154 Pa s and it falls well within acceptability for FFF three-dimensional printing (see [39,40]). This result confirms that PHBH is three-dimensional printable at the operating temperature of 175°C. It is also worth pointing out that the viscosity–shear rate relation (3.2) fails to describe the experimental curve shown in fig. 5 of [27], which is *pseudoplastic* (i.e. power-law fluid). This discrepancy may be due to the testing conditions, especially temperature, which is not specified.

### 3.2. Cytocompatibility

Cytocompatibility of biomaterials is essential for their successful application and, in particular, lack of cytotoxicity is a prerequisite in tissue engineering. In this analysis of PHBH cytocompatibility, cell viability and metabolic activity are considered acceptable when they scored beyond 80% of the blank control. Since cytotoxicity of the biopolymer may result from the material itself as well as from leachable compounds released from it, both extract and direct contact tests are performed.

**Figure 6.**
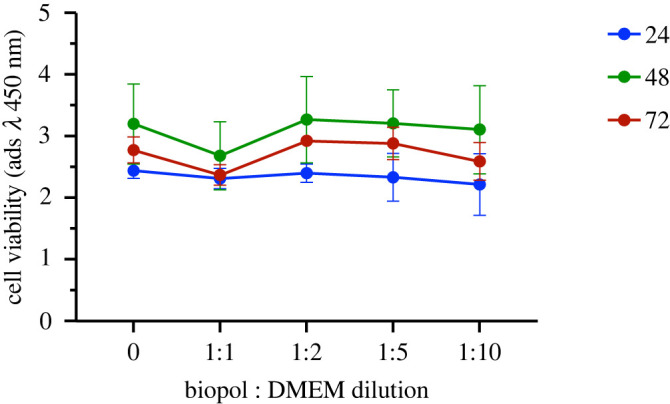
Extraction test: cell viability of balb-3T3 exposed to different dilution rates of biopolymer extraction medium at various times (time in h).

**Figure 7.**
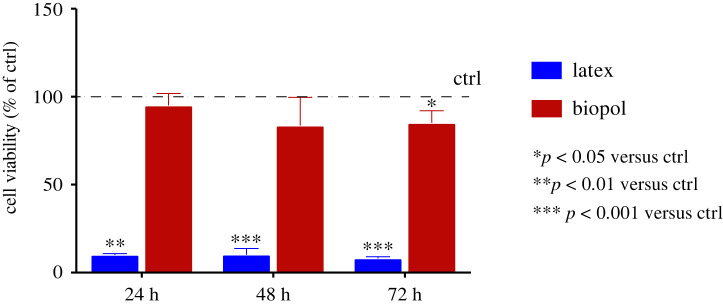
Extraction test: cell viability of balb-3T3 treated with pure biopolymer extraction medium at different time points, compared with the positive cytotoxic reference material.

Quantitative cytotoxicity analysis of biopolymer extracts diluted with DMEM 10%, 25%, 50% and 100% is performed by the metabolic activity assay at different incubation times. As shown in figure 6, the biopolymer did not show any cytotoxic activity both after 24 and 48 h of incubation time, in comparison with the control sample, at all dilution levels. At 48 h and 72 h incubation, we could observe a slight decrease of cell viability only at 1 : 1 dilution. Nonetheless, inhibition of cell viability of the 100% extraction medium did not reach significant values at 24 (%viability=96.0±6.8%), 48 (%viability=83.5±16%) and 72 h (%viability=85±6.9%). By contrast, latex induced massive cell death at all time steps (figure 7). It is interesting to compare figure 7 with fig. 9 of [27], where, remarkably, cell viability increases with respect to control for increasing polymer concentration.

**Figure 8.**
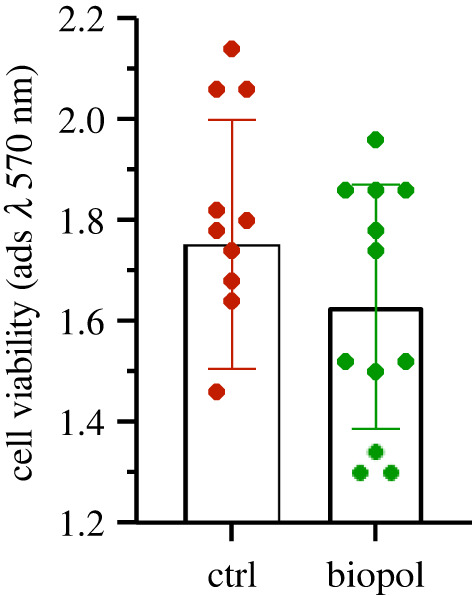
Direct contact test: cell metabolic activity of balb-3T3 cells exposed directly to the biopolymer for 24 h.

**Figure 9.**
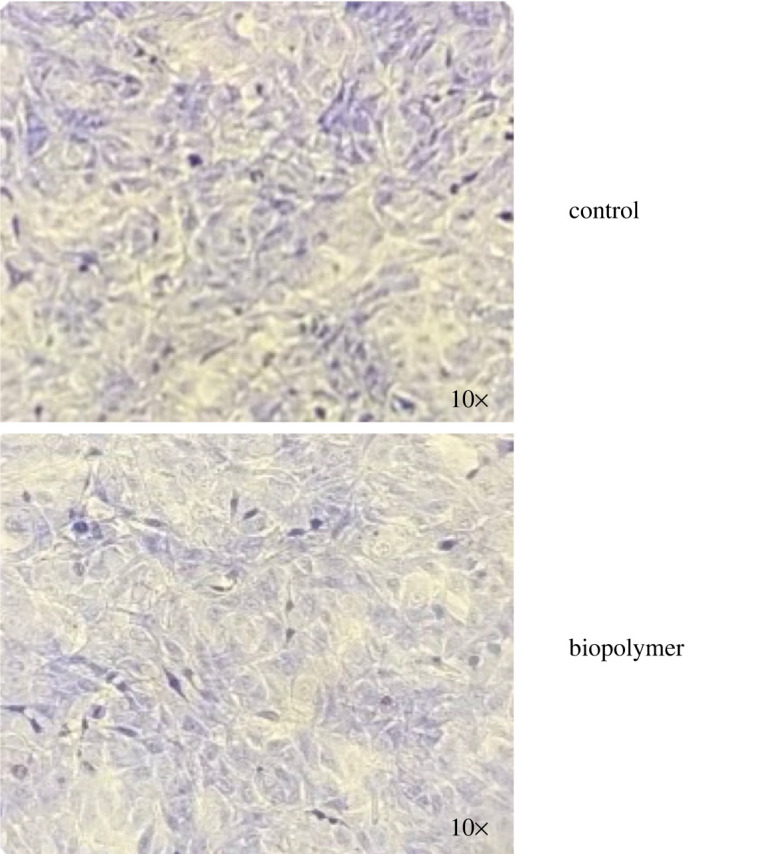
Direct contact test: micrograph of cells stained with GIMSA.

Lack of cytotoxicity is also supported by direct contact tests. Indeed, the metabolic activity of balb 3T3 cells in contact with the biopolymer, tested with MTT, is found to be statistically equivalent to that observed for cells cultured with the medium alone, as it is shown in the bar-chart of figure 8. Moreover, the number and morphology of the cells which were covered by the biopolymer are comparable to those observed in the cells culturing the plastic surface of the well (figure 9), which fact supports good cytocompatibility of the material.

### 3.3. Mechanical characterization

#### 3.3.1. Compression test on poly(3-hydroxybutyrate-*co*-3-hydroxyhexanoate) printed filled cubes

**Figure 10.**
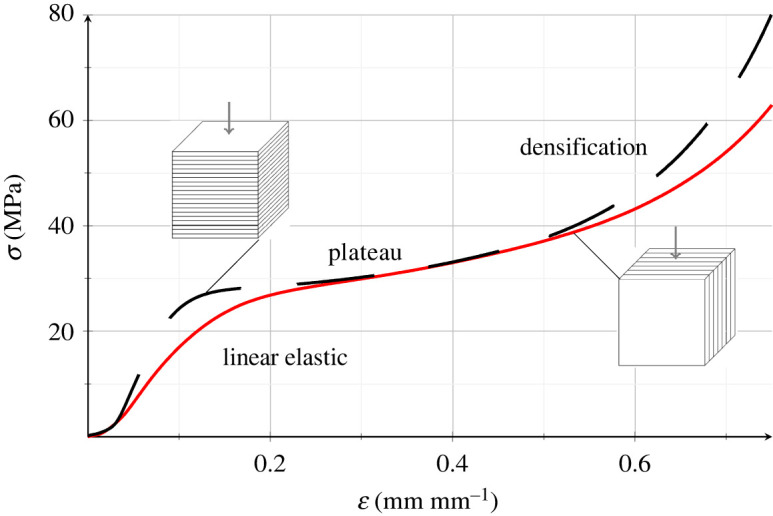
Experimental mean stress–strain curves of PHBH printed solid cubes under compression acting transversally to (black dashed line) and along (red solid line) the extrusion direction.

Figure 10 illustrates the experimental stress–strain curve for printed PHBH cubes, compressed in the longitudinal (black, dashed) and in the transversal (red, solid curve) directions. Curves express the mean across the relevant specimen group. They clearly exhibit three distinct regimes: an initial linear elastic tract, a stress plateau (plastic regime) and finally a region in which stress sharply increases (densification). The latter behaviour is typical of structures with porosities undergoing compressive displacement [23]. Indeed, as the applied strain increases, pores collapse and this leads to a densification process which takes place alongside rapid stress growth.

The mean elastic modulus and ultimate compressive strength are reported in table 2. As expected, cubes tested in the direction of extrusion show a stiffer response, with an elastic modulus that is 60% higher than that of transversally compressed specimens. The latter, in fact, are subjected to a delamination process, owing to the weak bond standing across adjacent deposition layers in the structure. This observation supports an anisotropic response. However, the difference in mechanical response is mainly restricted to the initial linear stage, and indeed the compressive strength at yield for the two testing directions differs by 19%. Conversely, the two curves almost overlap in the plateau and diverge again at densification.

**Table 2.** Mechanical properties of PHBH printed solid cubes undergoing compression along or across the extrusion direction (which is normal to the deposition plane).

specimen	characteristic	symbol	unit	value
longitudinal	modulus in compression	*E*_C_	MPa	364.8 ± 6.9
	compressive strength	*σ*_Y_	MPa	24.1 ± 0.1
transversal	modulus in compression	*E*_C_	MPa	226.7 ± 15.6
	compressive strength	*σ*_Y_	MPa	20.3 ± 1.6

**Figure 11.**
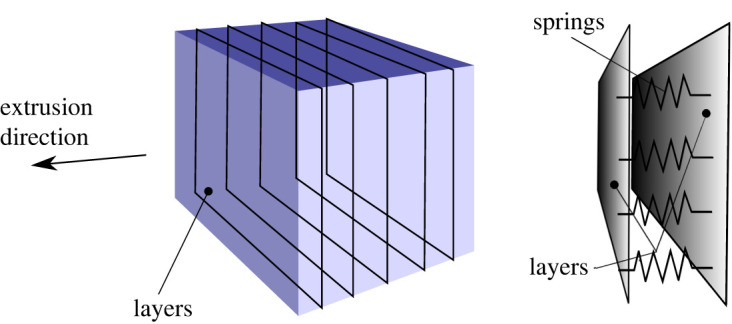
Deposition layers for a solid cube: deposition layers act as planes connected by springs.

This behaviour is explained by observing that the cube may be thought of as being composed of multiple adjacent layers which are connected by springs whose stiffness is larger in compression than in tension (figure 11). When the cube is tested along the extrusion direction, springs are compressed and the cube appears stiff. Conversely, when it is tested transversally to the extrusion direction, layers tend to separate (delamination) and it appears softer.

#### 3.3.2. Tensile test on poly(3-hydroxybutyrate-co-3-hydroxyhexanoate) dumb-bell shape specimens

**Figure 12.**
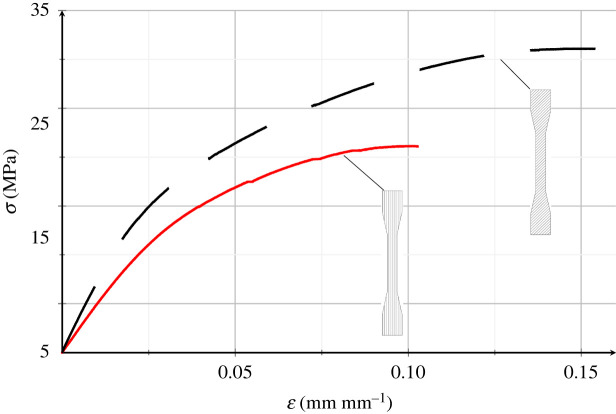
Experimental mean stress–strain curve for PHBH dumb-bell specimens under tension: diagonal (black, dashed curve) and longitudinal (red, solid curve) infill.

Figure 12 shows experimental stress–strain curves for tensile tests of dumb-bell specimens. Curves are mean across the relevant group for the deposition (infill) angle at 45° (black, dashed curve) and along (red, solid curve) the specimen axis, respectively, diagonal and longitudinal infill. Relevant mechanical properties are summarized in table 3. It is important to emphasize that such material parameters are sensibly smaller than those presented in table 7 of [27]. This difference reflects the importance of the printing process and of the scaffold geometry. It is precisely the role of these features which we attempt to describe in this section. The stress–strain curves reveal a linear elastic behaviour up to ϵ∼2.5%, corresponding to *σ* ∼ 15 MPa and *σ* ∼ 11 MPa for diagonal and longitudinal infill, respectively. The linear behaviour is followed by a nonlinear response wherein the stiffness progressively decays until fracture occurs. Although testing is carried out under displacement control, very little softening could be appreciated. It appears that the mechanical response is largely influenced by the printing process. Specimens printed with an infill angle of 45°, whose tensional properties are comparable to those reported in [27], exhibit elastic modulus in tension and ultimate stress 55% and 47% higher than the corresponding properties of longitudinal infilled specimens, respectively.

**Table 3.** Mechanical properties of PHBH dumb-bell specimens whose infill is printed along (longitudinal) or at 45° (diagonal) of the specimen longitudinal axis.

specimen	characteristic	symbol	unit	value
diagonal	modulus in tension	*E*_T_	MPa	750.3 ± 67.3
	maximum tensile stress	*σ*_B_	MPa	31.1 ± 2.4
	tensile strain at break	*ε*_B_	%	15.4 ± 2.0
longitudinal	modulus in tension	*E*_T_	MPa	485.6 ± 0.1
	maximum tensile stress	*σ*_B_	MPa	21.1 ± 2.7
	tensile strain at break	*ε*_B_	%	10.3 ± 0.4

**Figure 13.**
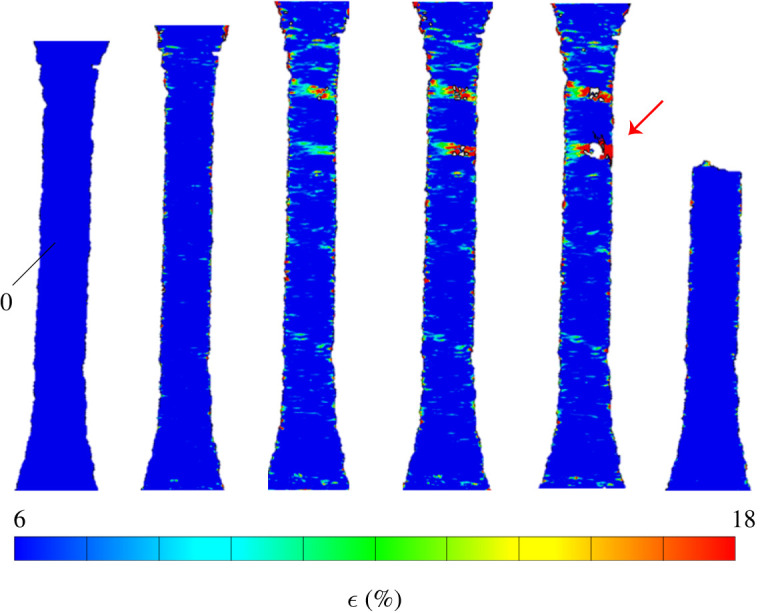
Snapshots of the strain field as tensile test evolves, from left to right, until failure occurs. Defects clearly appear as strain concentrations in the strain map (DIC analysis). A red arrow indicates the defect that develops into a visible crack and ultimately leads to failure.

**Figure 14.**
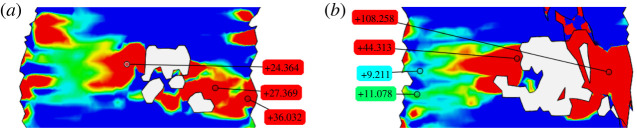
Detail of the strain field [%] in the neighbourhood of the top (*a*) and bottom (*b*) defect observed in figure 13 (DIC analysis: white regions indicate failure to correlate). The strain concentration in the bottom defect is three to four times bigger than that given by the top defect.

During tensile testing, DIC was carried out to determine the strain level prior to failure. Figure 13 shows the strain field at different time steps for a typical analysis, from the reference (left) to the failed configuration (right), where the specimen breaks in two (only the bottom piece is illustrated). The deformation builds up gradually in the material except in special regions (defects), where it spikes. The picture shows that two transversal strain concentration zones develop in the specimen, one after the other. Yet only one defect (marked by a red arrow in figure 13) develops into a macro-crack and ultimately leads to failure. It is emphasized that only this crack could be observed at the end of the test. Magnified views of the strain field near the defects and approaching failure are reported in figure 14. It is clear that the lower defect leading to failure develops exceedingly high levels of local deformation, in excess of 100% (figure 14*b*). By contrast, the top defect, which emerges first but never develops into a macroscopic crack, shows significantly lower strain levels, under 40% (figure 14*a*). Further analysis suggests that failure occurs for ultimate deformation levels that, locally, sit in the range 70%–100%. Comparing this result with the mean ultimate strain at failure (which is around 15%, according to table 3), we see that defects act as very important stress concentration points. They are indeed present in the printed structure and greatly magnify the local strain. Although this is not investigated here, it is a matter of great importance to relate the presence of defects to the quality and to the parameters of the printing process.

**Figure 15.**
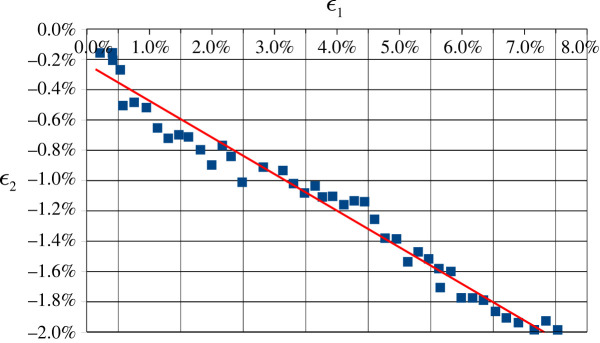
DIC extracted data points for the longitudinal *ε*_1_ and transversal *ε*_2_ deformation at different time steps along the traction test for the longitudinal infill: Poisson’s ratio is obtained as (the opposite of) the angular coefficient of the linear fit (red, solid line).

DIC analysis is also exploited to determine Poisson’s ratio as the angular coefficient of the linear fit in the (*ε*_1_, −*ε*_2_) plane. Figure 15 shows this analysis for a typical longitudinal infill sample: data are well clustered around the linear fit with a coefficient of determination *R*^2^ = 0.9660. The average Poisson’s ratio across different specimens and infills is reported in table 4 and shows that the material is far from being incompressible.

**Table 4.** Effective mechanical properties adopted in the FEA of printed scaffolds in the linear and nonlinear regime. For the nonlinear regime, the compressible Mooney–Rivlin model (2.4) is chosen.

characteristic	symbol	unit	value
Poisson ratio	*ν*	—	0.28
Young modulus	*E*_C_	MPa	270
bulk modulus	*K*	MPa	205
shear modulus	*G*	MPa	140
1st invariant coeff.	*c*_1_	MPa	250
2nd invariant coeff.	*c*_2_	MPa	−180

#### 3.3.3. Compression test on poly(3-hydroxybutyrate-*co*-3-hydroxyhexanoate) printed scaffolds

**Figure 16.**
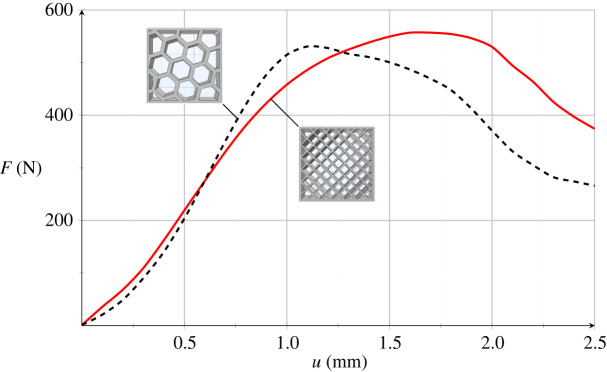
Experimental mean strength curve for scaffolds with cubic (red, solid) and honeycomb (black, dashed curve) porosity.

Figure 16 displays the experimental strength curve under uniaxial compression of PHBH printed scaffolds with honeycomb (black dashed line) and cubic (red solid line) porosity. As usual, curves are the mean within the relevant porosity group. Although the behaviour is complex, we still recognize a linear elastic tract up to *u* ∼ 0.8 mm (which corresponds to ϵ=4%, F∼440 N and F∼400 N for honeycomb and cubic porosity, respectively), followed by a nonlinear response which eventually develops into a distinct softening branch. This behaviour clearly differs from that illustrated in figure 10 for dumb-bell specimens in light of the complex mechanism of porosity collapse.

**Figure 17.**
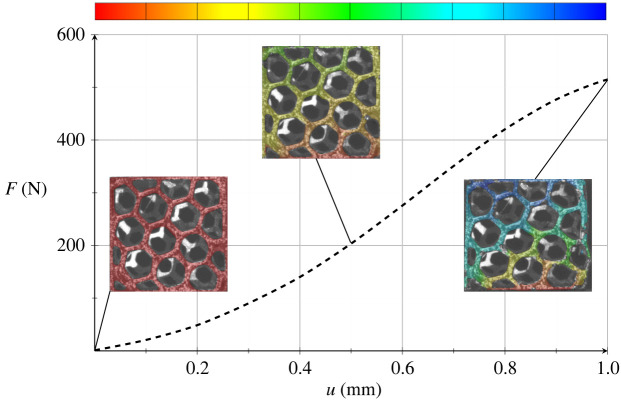
Scaffold with honeycomb porosity: mean strength curve and displacement field at three displacements, namely 0 mm, 0.5 mm and 1 mm.

**Figure 18.**
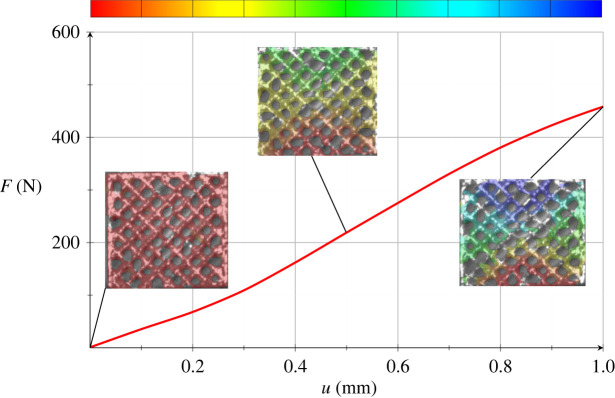
Scaffold with square porosity: mean strength curve and displacement field at three displacements, namely 0 mm, 0.5 mm and 1 mm.

This may be illustrated by figures 17 and 18 that present the displacement field measured by DIC on the scaffold surface at three load steps, corresponding to the applied displacement 0 mm, 0.5 mm (ϵ=2.5%) and 1 mm (ϵ=5%). We clearly see the early collapse of the scaffold bounding wall, especially for the honeycomb porosity. The entailing deformation behaviour turns more and more asymmetric and it is compared against the numerical simulation of the scaffold compression test, as presently illustrated.

#### 3.3.4. Finite-element analysis

In order to fully determine the nonlinear effective properties of the scaffolds, numerical simulations with a commercial FE software (COMSOL Multiphysics^®^) are performed, in an attempt to reproduce the deformation field measured through DIC. Variable-order and variable-step-size backward differentiation formulae (BDF) are selected for the time integration scheme of a uniform CST discretized mesh that is constructed on top of the specimen camera image (as opposed to the feed to the printing machine, which may not correctly represent the printed scaffold). It is emphasized that the nonlinear response (2.4) is adopted, wherein *c*_1_ and *k* are measured directly in the linear regime. Consequently, FE-model-to-data fitting is employed to assess only the parameter *c*_2_, which mitigates any concern of ill-convergence and non-uniqueness. In fact, for small *ε*, the computed curve is bound to rest close to the linear approximation regardless of *c*_2_, whose role is mainly to locate the onset of softening (that occurs at ϵ∼4%). It is also worth mentioning that this robust approach comes at the price of greatly restricting our fitting capability, especially in the case of the honeycomb porosity. Accordingly, this may be regarded as a minimal approach, which may be successively improved.

**Figure 19.**
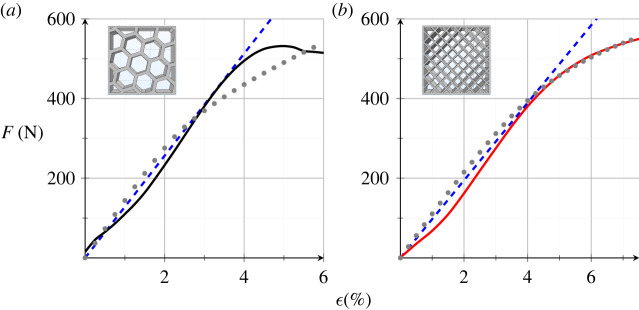
Comparison between experimental data (black and red solid curve), linear (blue dashed) and nonlinear (grey dotted) numerical simulations.

Figure 19 compares experimental results (solid curve) with numerical simulations, in the linear (blue, dashed) and nonlinear (grey, dotted curve) regime. Honeycomb (figure 19*a*) and cubic (figure 19*b*) porosities are considered. Simulations are carried out with the parameter set listed in table 4. It appears that the linear regime holds well up to ϵ=4% and it is followed by rapid stiffness loss, which is probably due to local buckling of the bounding wall. While square porosity is well captured by numerical simulations, the same cannot be said for honeycomb porosity, whose sudden stiffness loss into a perfect plastic regime eludes Mooney–Rivlin.

**Figure 20.**
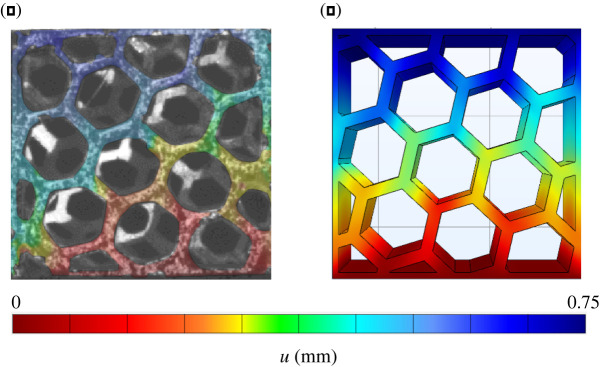
Comparison between measured (DIC) (*a*) and calculated (FE) (*b*) displacement field for scaffolds with honeycomb porosity.

**Figure 21.**
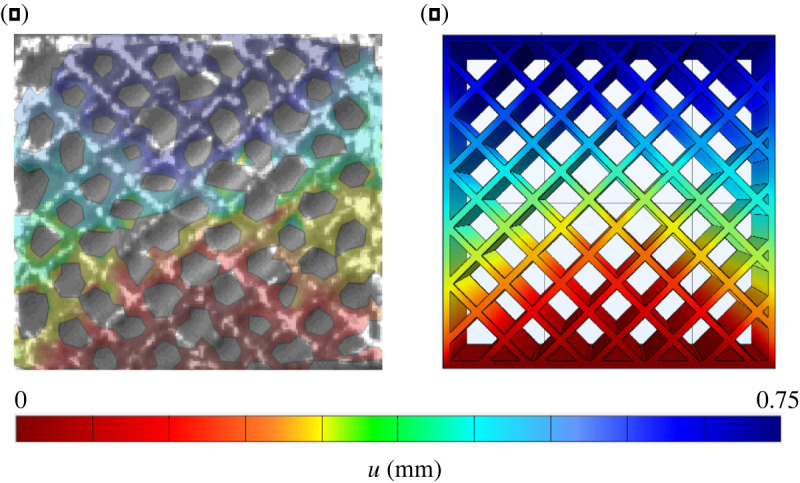
Comparison between measured (DIC) (*a*) and calculated (FE) (*b*) displacement field for scaffolds with cubic porosity.

Nonetheless, qualitative comparison reveals remarkable similarity in terms of the displacement field between experimental data and simulations. Figures 20 and 21 present this comparison at ϵ=3,75%, that is at the end of the linear regime. Unexpectedly, this similarity extends to the full displacement field and to both scaffold porosities.

## 4. Conclusion

In this work, thermal, rheological and cytotoxicity properties of three-dimensional FFF printed PHBH scaffolds have been investigated. It is found that PHBH possesses suitable characteristics for its use in three-dimensional FFF at 175°C. Besides, the material is biocompatible and shows no evidence of cytotoxicity in both direct and extraction tests. To the authors’ best knowledge, effective mechanical properties of PHBH printed scaffolds have been determined in the linear and nonlinear regime for the first time. This knowledge, which is crucial for the successful design of tissue supports, is here mainly obtained by direct measurement, while model fitting is reverted to for determining one nonlinear modulus only. This approach warrants robustness and uniqueness of the moduli. Since the material response is heavily dependent on the printing process, on the scaffold porosity and on the deposition sequence, a homogeneous isotropic model, albeit nonlinear, should be only regarded as a first approach approximation. Yet, comparing numerical simulations of the compression tests with DIC experimental data suggests that effective properties are able to reproduce the deformation field pattern with surprising accuracy. Indeed, the compressible Mooney–Rivilin material model proves especially effective for the cubic porosity. The role of local defects in inducing high strain gradients, which eventually develop into cracks and lead to failure, is experimentally documented. Failure initiation is associated with local deformation peaks in the order of 100% strain. Conversely, we observe that the material is able to withstand local strain levels up to 40% without developing cracks. Interestingly, this strain level is significantly higher than the average ultimate tensile deformation obtained from the stress–strain curve (about 15%). Results can be successfully employed for the correct design of biocompatible, reabsorbable FFF printed scaffolds for tissue regeneration, bone implants and healing and damage reconstruction in the presence of mechanical strain. In particular, PHBH printed scaffolds exhibit significant potential for implants in trabecular bone, whose compressive modulus lies in the range 50–500 MPa [41], and it is therefore compatible with that observed for the scaffolds.

## Data Availability

Raw data are uploaded as electronic supplementary material [42].
